# Fludarabine-mediated suppression of the excision repair enzyme ERCC1 contributes to the cytotoxic synergy with the DNA minor groove crosslinking agent SJG-136 (NSC 694501) in chronic lymphocytic leukaemia cells

**DOI:** 10.1038/sj.bjc.6603853

**Published:** 2007-06-19

**Authors:** C Pepper, H Lowe, C Fegan, C Thurieau, D E Thurston, J A Hartley, P Delavault

**Affiliations:** 1 Department of Haematology, School of Medicine, Cardiff University, Heath Park, Cardiff, UK; 2 Cancer Research UK Drug-DNA Interactions Research Group, Department of Oncology, University College London, London, UK; 3 Ipsen, Research & Development, Paris, Cedex 16, France; 4 Department of Pharmaceutical and Biological Chemistry, Cancer Research UK Gene Targeted Drug Design Research Group, School of Pharmacy, University of London, Brunswick Square, London, UK

**Keywords:** SJG-136, PBD dimer, DNA crosslinking, chronic lymphocytic leukaemia, apoptosis, synergy

## Abstract

In this study, we set out to establish whether fludarabine could enhance the DNA interstrand crosslinking capacity of SJG-136 in primary human chronic lymphocytic leukaemia (CLL) cells and thereby offer a rationale for its clinical use in combination with SJG-136. SJG-136 rapidly induced DNA crosslinking in primary CLL cells which was concentration-dependent. Further, the level of crosslinking correlated with sensitivity to SJG-136-induced apoptosis (*P*=0.001) and higher levels of crosslinking were induced by the combination of SJG-136 and fludarabine (*P*=0.002). All of the samples tested (*n*=40) demonstrated synergy between SJG-136 and fludarabine (mean combination index (CI)=0.54±0.2) and this was even retained in samples derived from patients with fludarabine resistance (mean CI=0.62±0.3). Transcription of the excision repair enzyme, ERCC1, was consistently increased (20/20) in response to SJG-136 (*P*<0.0001). In contrast, fludarabine suppressed ERCC1 transcription (*P*=0.04) and inhibited SJG-136-induced ERCC1 transcription when used in combination (*P*=0.001). Importantly, the ability of fludarabine to suppress ERCC1 transcription correlated with the degree of synergy observed between SJG-136 and fludarabine (*r*^2^=0.28; *P*=0.017) offering a mechanistic rationale for the synergistic interaction. The data presented here provides a clear indication that this combination of drugs may have clinical utility as salvage therapy in drug-resistant CLL.

B-cell chronic lymphocytic leukaemia (CLL) is an incurable malignancy with a variable clinical course (Bosch and Montserrat, 2002). Outcome for advanced-stage CLL patients is poor, with an expected median survival of 3 years (Lee *et al*, 1987). Although the introduction of purine nucleoside analogues (Zhu *et al*, 2004) and monoclonal antibody therapies (Byrd *et al*, 2001; Keating *et al*, 2002), either as single agents or in combination (Byrd *et al*, 2003; Kay, 2006), has led to significantly increased response rates, none of these options is curative. Consequently, there is a need to identify new agents for the treatment of CLL, particularly agents that have the potential to complement existing therapeutic options and overcome drug resistance.

SJG-136 (NSC 694501, BN2629, SG2000) is a novel DNA crosslinking agent that binds in a sequence-specific manner the minor groove of the helix (Gregson *et al*, 2001; Hartley *et al*, 2004). It is structurally novel compared to other clinically used DNA crosslinking agents and has exhibited a unique multilog differential pattern of activity in the NCI 60 cell line screen (Hartley *et al*, 2004). SJG-136 also displayed potent activity against several human tumours *in vivo* (Alley *et al*, 2004), and phase I clinical trials against both solid tumours and haematological malignancies are currently underway. We have previously shown that SJG-136 is extremely cytotoxic to CLL cells *in vitro*, exhibiting LD_50_ values more than 2 logs lower than fludarabine, the current treatment of choice for this condition (Pepper *et al*, 2004). Furthermore, SJG-136 exhibited differential cytotoxicity since it was less effective at inducing apoptosis in normal B- and T-lymphocytes derived from CLL patients.

The formation of drug-induced crosslinks between two complementary strands of DNA, interstrand crosslinking (ICL), is considered to be critical to the activity of bifunctional alkylating agents, and there is clear evidence that their formation and subsequent persistence correlates to *in vitro* cytotoxicity (Sunters *et al*, 1992). In this context, it has been previously demonstrated that fludarabine can inhibit excision and repair of ICL and by so doing contributes to the synergy observed between fludarabine and cisplatin (Yang *et al*, 1995; Li *et al*, 1997). This inhibition of ICL repair was associated with the inhibition of the nucleotide excision repair enzyme, ERCC1. In addition, a recent report clearly demonstrated a role for ERCC1 in the repair of SJG-136-induced DNA ICLs (Clingen *et al*, 2005). Therefore, this present study was designed to investigate whether fludarabine-mediated inhibition of ERCC1-mediated DNA repair could positively impact on SJG-136 ICL patterns and enhance the resulting cytotoxicity of SJG-136 in CLL cells.

## MATERIALS AND METHODS

### Patients' cells and clinical details

Peripheral blood samples from 40 patients with CLL were obtained with the patients' written informed consent in accordance with the conditions for ethical approval granted by the Southeast Wales Research Ethics Committee (no. 02/4806). Chronic lymphocytic leukaemia was defined by clinical criteria, and staging was based on the Binet system (Binet *et al*, 1981). None of the previously treated patients had received therapy for at least 1 month before the study. V_H_ gene mutational status, CD38 expression and ZAP-70 expression was available for all of the patients under investigation. The clinical characteristics of the patient cohort are summarised in Table 1.

### Primary CLL cell-culture conditions

Freshly isolated peripheral blood lymphocytes (1 × 10^6^ ml^−1^) were cultured in RPMI medium (Invitrogen, Paisley, UK), supplemented with 100 U ml^−1^ penicillin, 100 *μ*g ml^−1^ streptomycin and 10% fetal calf serum. Lymphocytes were incubated at 37°C in a humidified 5% carbon dioxide atmosphere in the presence of SJG-136 (10^−10^–10^−7^ M), fludarabine (10^−7^–10^−5^ M) or both drugs in combination at fixed molar ratio of 1 : 100 (SJG-136 : fludarabine). The optimal ratio for synergy between the two agents was determined experimentally (data not shown). In addition, control cultures were carried out to which no drug was added. All experiments were performed in duplicate.

### Single-cell gel electrophoresis assay

The level of DNA ICL was determined using a modified single-cell gel electrophoresis (comet) assay (Hartley *et al*, 1999; Spanswick *et al*, 2002). Each sample was split into two for the separate analysis of single-strand breaks and DNA ICL. The sample for crosslink analysis was irradiated (12.5 Gy, 2.35 Gy min^−1^) immediately before analysis to deliver a fixed number of random DNA-strand breaks. After embedding cells in 1% agarose the cells were lysed for 1 h in lysis buffer and then washed for 1 h in distilled water. Slides were then incubated in alkali buffer for 45 min followed by electrophoresis in the same buffer for 25 min.

After drying, the slides were stained with propidium iodide for 30 min then rinsed in distilled water. Images were captured using an online CCD camera and analysed using Komet Analysis software (Kinetic Imaging, Liverpool, UK). For each duplicate slide, 25 cells were analysed. The tail moment for each image was calculated using the Komet Analysis software as the product of the percentage DNA in the comet tail and the distance between the means of the head and tail distributions, based on the definition of Olive *et al* (1990). Crosslinking was expressed as the percentage decrease in tail moment compared to irradiated controls and was calculated using the formula

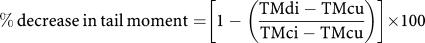
 
where TMdi is the tail moment of drug-treated irradiated sample, TMcu the tail moment of untreated, unirradiated control, and TMci the tail moment of untreated, irradiated.

In combination studies of SJG-136 and fludarabine, the following formula was used:

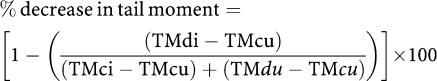
 
where TMdu is the tail moment of drug-treated, unirradiated samples to take into account any extra strand breaks produced by fludarabine.

### Measurement of *in vitro* apoptosis

Cultured cells were harvested by centrifugation. Apoptosis was assessed by dual-colour immunofluorescent flow cytometry as described previously (Vermes *et al*, 1997; Pepper *et al*, 2003).

### Assessment of synergy between SJG-136 and fludarabine

Cells were incubated with various concentrations of fludarabine, SJG-136 and fludarabine in an optimised fixed-ratio combination with SJG-136 for 48 h at 37°C. Dose–response curves were plotted for each individual drug and drug combination, and the curves were analysed using the median effect method to determine the degree of synergy (Chou and Talalay, 1984). These experiments were conducted in two groups of samples, that is those derived from patients who had demonstrated either *in vitro* and/or clinical resistance to fludarabine (20 patients) and CLL samples that retained sensitivity to fludarabine (20 patients) to assess the drug interactions in each cohort.

### RT–PCR analysis of ERCC1 transcription

Total RNA was extracted from 3 × 10^7^ CLL lymphocytes using RNeasy spin columns according to the manufacturer's instructions (Qiagen, Crawley, UK). One microgram of total RNA was then reverse-transcribed using a Clontech first-strand cDNA synthesis kit (Clontech, Between Towns, Cowley, Oxford, UK). Subsequently, real-time PCR was performed using ERCC1-specific primers CCGCCAGCAAGGAAGAAATT (forward) and TTACGTCGCCAAATTCCCAG (reverse) and S14 (house-keeping gene) primers GGCAGACCGAGATGAACTCT (forward) and CCAGGTCCAGGGGTCTTGGT (reverse) in PCR buffer containing 50 nM of each primer, 4.0 mM MgCl_2_ cDNA template and FastStart SYBR Green I DNA master mix (Roche Diagnostics, Welwyn Garden City, Hertfordshire, UK) in a 10 *μ*l final volume. Each reaction was cycled 45 times consisting of a 10 s denaturation (95°C), 5 s annealing (60°C) and 20 s extension (72°C). To confirm the amplification specificity the PCR products were subjected to melting-curve analysis. The S14:ERCC1 transcription ratio was calculated from the crossing points of each gene.

### Statistical analysis

The data obtained in these experiments were subjected to the Kolmorgorov–Smirnov test of normality; all the data sets tested were shown to be Gaussian. Consequently, statistical analyses were performed using the paired Student's *t*-test. Correlation coefficients were calculated from least-squares linear regression plots, and drug sensitivity was evaluated using nonlinear regression and line of best-fit analysis of the sigmoidal dose–response curves. All statistical analyses were performed using Graphpad Prism 3.0 software (Graphpad Software Inc., San Diego, CA, USA). Synergy was assessed using Calcusyn software (Biosoft, Cambridge, UK).

## RESULTS

### SJG-136-induced DNA ICL

**Figure 1:**
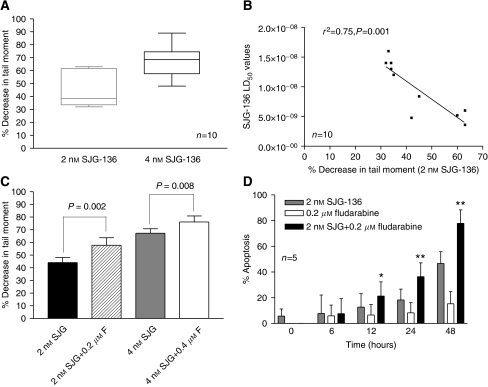
Percentage decrease in comet tail moment in CLL samples treated with SJG-136±fludarabine for 24 h. The decrease in tail moment reflects an increase in DNA ICL. (**A**) Ability of SJG-136 to produce DNA ICL in a concentration-dependent manner in the samples tested. (**B**) The degree of DNA crosslinking correlated with the cytotoxic effects of SJG-136 *in vitro*. (**C**) Effect of the combination of SJG-136 and fludarabine resulting in increased DNA crosslinking. (**D**) Apoptosis was markedly enhanced in a time-dependent manner in samples treated with the combination of SJG-136 and fludarabine when compared to either drug alone. The increase in apoptosis was evident after 12 h (^*^P<0.05) and became more marked after 24 and 48 h (^**^P<0.0001).

The level of DNA ICL was determined using a modification of the comet assay. Figure 1A shows the mean (±s.d.) percentage decrease in tail moment in CLL samples derived from 10 patients following exposure to 2 and 4 nM SJG-136 for 24 h. A decrease in tail moment has previously been shown to reflect an increase in DNA ICL (Hartley *et al*, 1999). Therefore, SJG-136 was shown to be a potent ICL agent in all the CLL samples tested with a mean concentration of SJG-136 that caused a 50% decrease in tail moment of approximately 2.6 nM. Importantly, the degree of DNA crosslinking was correlated with *in vitro* sensitivity to SJG-136 as determined by measurement of apoptosis using Annexin V (Figure 1B) suggesting that ICL formation was pivotal to the cytotoxic effects of SJG-136.

The addition of fludarabine at a fixed molar ratio of 1 : 100 (SJG-136 : fludarabine) resulted in increased DNA crosslinking in the majority of samples tested (9/10) with an average increase of 32.8% at the 2 nM concentration of SJG-136. The comparisons of DNA crosslinking at 2 nM and 4 nMSJG-136±fludarabine are shown in Figure 1C. We also performed time-course experiments using both the comet assay and the Annexin V assay to establish the kinetics of the DNA crosslinking and apoptosis induction in primary CLL cells. In keeping with previous findings (Clingen *et al*, 2005), SJG-136 rapidly induced DNA crosslinks and these were maximal after 24 h exposure (data not shown). Apoptosis was also induced in a time-dependent manner with clear evidence of enhanced apoptotic cell killing in the cultures exposed to the combination of SJG-136 and fludarabine (Figure 1D). By 12 h the combination was statistically more cytotoxic than SJG-136 alone (*P*=0.04) and by 24 h this was even more marked (*P*<0.0001). This enhanced cytotoxicity appeared greater than the summative apoptotic effects of the two agents alone suggesting a synergistic interaction.

### SJG-136 and fludarabine synergise in primary CLL cells

**Figure 2:**
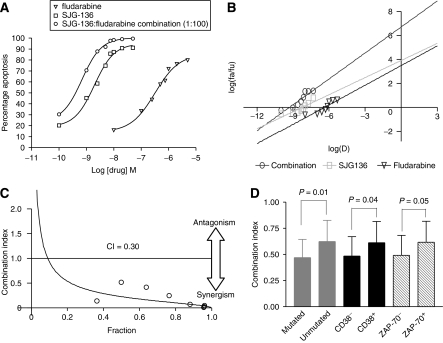
Synergistic cytotoxicity produced by SJG-136 and fludarabine in primary CLL cells. (**A**) Dose–response curves were generated for CLL cells treated with SJG-136 and/or fludarabine. The cytotoxicity was quantified using an Annexin V/propidium iodide assay. (**B**) Median-effect plot. The cells were treated for 48 h with SJG-136 (1.0, 2.5, 5.0, 7.5, 10.0, 20.0 or 50 nM), fludarabine (0.1, 0.25, 0.5, 0.75, 1.0, 2.0 or 5.0 *μ*M), or both at a fixed molar ratio of 1 : 100. The median-effect plot was constructed using Calcusyn software where Fa is the fraction affected and Fu the fraction unaffected. The fraction-affected CI plot was constructed by computer analysis of the data in (**B**) using the conservative isobologram (**C**). Combination index values of <1 occurred at a wide range of inhibition levels, indicating that synergy was produced by the combination. (**D**) Analysis of prognostic subsets revealed that there was diminished synergy in samples derived from patients with unmutated V_H_ genes, high CD38 expression and high ZAP-70 expression. However, this is likely to be the result of a preponderance of these samples demonstrating *in vitro* or *in vivo* fludarabine resistance.

The cytotoxic synergy between SJG-136 and fludarabine was assessed in all 40 samples. Figure 2A shows the dose–response of CLL cells derived from one patient treated with various concentrations of SJG-136 and fludarabine as single agents and in combination. The combination of the two drugs was significantly more cytotoxic than either single agent. Furthermore, Figure 2B shows that the slopes of the median-effect plots for the single drugs and the combination were not parallel, suggesting that the two agents had different modes of action. Therefore, the equation for the conservative isobologram was used to calculate the combination index (CI) shown in the fraction-affected CI plot (Figure 2C). All 40 samples showed synergy between the two drugs at a fixed molar ratio of 1 : 100 (SJG-136 : fludarabine) with a mean CI of 0.54±0.2. Combination index values less than 1 denote synergy, with the smallest values representing the greatest degree of synergy. Although the degree of synergy was significantly diminished in patient samples defined as fludarabine resistant when compared with fludarabine-sensitive samples (*P*=0.03), synergy was still demonstrated in all these samples (mean CI=0.62±0.3). The drug-sensitivity data for fludarabine-sensitive and fludarabine-resistant patient groups are summarised in Table 2. Analysis of the degree of synergy, as defined by CI values, in prognostically distinct subsets of CLL patients revealed that patients with unmutated V_H_ genes, high CD38 expression and high ZAP-70 expression all showed significantly reduced synergy (Figure 2D). However, this observation is likely to be caused by a greater proportion of these subsets having either *in vitro* or *in vivo* fludarabine resistance.

### Inhibition of transcription of the excision repair enzyme ERCC1 by fludarabine

**Figure 3:**
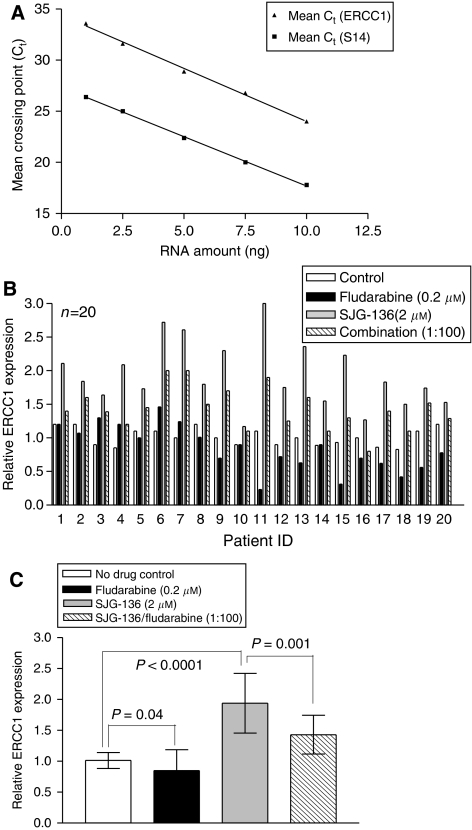
Real-time quantitative PCR for ERCC-1 expression. Initial amount of cDNA in each reaction was plotted against the *C*_t_ values for the house-keeping gene S14 and the target gene ERCC-1, and standard curves plotted. (**A**) The correlation coefficient for each curve was 0.99 suggesting that the amplification efficiencies for each reaction were similar. Data are representative of three independent experiments. (**B**) There was marked interpatient variability in ERCC1 transcription following exposure to fludarabine, SJG-136 or a combination of both agents. (**C**) The mean change in ERCC1 transcription (±) s.d. normalised to the control (no drug) sample for all 20 patients revealed that fludarabine suppressed ERCC1 transcription both alone and in combination with SJG-136.

The consistent demonstration of synergy between SJG-136 and fludarabine in this study led us to consider the possible mechanism that potentiated this effect. It has previously been demonstrated that fludarabine can inhibit DNA repair mechanisms and thereby synergise with cisplatin in the human chronic myeloid leukaemic K562 cell line (Yang *et al*, 1995; Li *et al*, 1997). More recently, Clingen *et al* (2005) reported that the XPF-ERCC1 endonuclease contributes to the repair of SJG-136-induced minor groove DNA ICL in Chinese hamster ovary (CHO) cells. Therefore, we hypothesised that fludarabine may suppress ERCC1 transcription and thereby enhance the cytotoxic effects of SJG-136. We first analysed serial dilutions of cDNA derived from CLL patient RNA to compare the efficiency of the PCR amplification reactions for ERCC1 and S14 (Figure 3A). It could be demonstrated in three independent experiments that both PCR ran with almost identical amplification efficiency, which is a prerequisite for using the change in crossing point (Δ*C*_t_) method for relative quantitation (Winer *et al*, 1999). To take into account the variation in total RNA introduced into each reaction, transcripts of S14 were quantified as an endogenous RNA control, and each sample was normalised on the basis of its S14 content. In addition, the relative amounts of ERCC1 and S14 were also normalised to a control sample (untreated cells). There was interpatient variability in terms of the magnitude of change in ERCC1 transcription following exposure to both fludarabine and SJG-136 (Figure 3B). However, fludarabine suppressed ERCC1 transcription in the majority of samples (15/20) and SJG-136 caused an increase in ERCC1 transcription (20/20) when compared to controls. Analysis of all 20 patients studied (Figure 3C) confirmed that fludarabine significantly suppressed ERCC1 transcription (*P*=0.04), SJG-136 significantly induced ERCC1 transcription (*P*<0.0001) and the combination of fludarabine and SJG-136 resulted in a suppression of ERCC1 transcription when compared to SJG-136 alone (*P*=0.001).

### Suppression of ERCC1 transcription correlates with the degree of synergy observed with SJG-136

**Figure 4:**
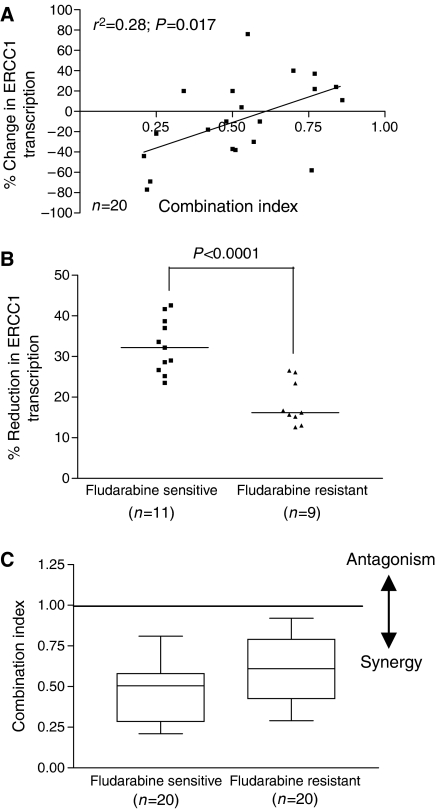
Relationship between change in ERCC1 transcription and the degree of synergy between SJG-136 and fludarabine. (**A**) The percentage change in ERCC1 transcription induced by fludarabine correlated with the degree of synergy observed between SJG-136 and fludarabine as measured by CI. (**B**) The fludarabine-sensitive samples showed a significant increased ability to suppress ERCC1 transcription when compared with the fludarabine-resistant subset (*P*<0.0001). (**C**) This resulted in significantly diminished synergy in the fludarabine-resistant subset (*P*=0.03).

We next investigated whether fludarabine-mediated suppression of ERCC1 transcription was mechanistically relevant to the synergy observed between SJG-136 and fludarabine. To do this, we compared the percentage reduction in ERCC1 transcription induced by fludarabine in each patient sample with the degree of synergy between SJG-136 and fludarabine as measured by the CI. Figure 4A shows the positive correlation between these two parameters suggesting a mechanistic link between increased suppression of ERCC1 transcription and enhanced synergy between the two drugs. Further evidence for this association was derived from subset analysis of the fludarabine-sensitive and fludarabine-resistant patient groups. The diminished degree of synergy demonstrated in the fludarabine-resistant subset was strongly associated with the reduced capacity of fludarabine to suppress ERCC1 transcription (Figure 4B and C). However, the retention of synergy in these samples indicates that the mechanisms that underpin the synergistic interaction between fludarabine and SJG-136 are more complex than the transcriptional regulation of one excision repair enzyme.

## DISCUSSION

In this study, we set out to establish whether fludarabine could enhance the DNA ICL capacity of SJG-136 in primary human CLL cells and thereby offer a rationale for its clinical use in combination with SJG-136. Using a modified comet assay to measure DNA crosslinking, we demonstrated that SJG-136 is an efficient, concentration-dependent, ICL-inducing agent in primary CLL cells. Importantly, the level of ICL correlated with sensitivity to SJG-136, suggesting a causal link between this critical lesion and cytotoxicity. Time course analysis revealed that the percentage of SJG-136-induced ICL was similar at both 24 and 48 h (data not shown). These results are in accord with those described by Hartley *et al* (2004), who showed that SJG-136-induced DNA ICL persisted both *in vitro* and *in vivo* when compared to those produced by conventional major groove binding drugs inferring that the kinetics of the repair of SJG-136-induced DNA crosslinking are very slow. However, co-incubation of SJG-136 and fludarabine resulted in an enhanced DNA crosslinking profile in the majority of the CLL cell samples tested (9/10), indicating that the combination may be more cytotoxic than SJG-136 alone.

Consequently, the next question that we wanted to address was to establish whether fludarabine could synergise with SJG-136 to induce more effective cell killing in primary human CLL cells. All of the samples tested (*n*=40) at the experimentally optimised 1 : 100 fixed molar ratio (SJG-136 : fludarabine) demonstrated a high level of synergy (mean CI=0.54±0.2). Importantly, this synergistic relationship, albeit diminished, was retained even in samples derived from patients with either *in vitro* or *in vivo* fludarabine resistance (mean CI=0.62±0.3). The fludarabine-resistant subset was characterised by a predominance of samples derived from patients with poor prognostic markers; unmutated V_H_ genes (16/20), high CD38 expression (14/20) and high ZAP-70 expression (12/20). As a result, subset analysis of these groups revealed statistically diminished synergy between SJG-136 and fludarabine in all of these poor prognostic subsets. However, synergy was retained in these poor risk groups, suggesting that the combination of SJG-136 and fludarabine may be particularly useful in these patients as well as in the salvage of fludarabine-resistant disease.

Previous work has shown that fludarabine can inhibit the repair of cisplatin- and oxaliplatin-induced DNA crosslinking (Yang *et al*, 1995; Li *et al*, 1997; Moufarij *et al*, 2006). Importantly, this appears to be more marked in CLL cells when compared to normal lymphocytes (Moufarij *et al*, 2006). In addition, Clingen *et al* (2005) demonstrated that sensitivity to SJG-136 was dependent to some extent on ERCC1 expression in CHO cells. To determine whether ERCC1 was induced by SJG-136 in primary CLL cells, we performed real-time RT-PCR on RNA extracted from CLL cells exposed to SJG-136, fludarabine and both drugs in combination. All of the samples tested (20/20) showed a marked increase in ERCC1 transcription in response to SJG-136 when compared to untreated control samples. In contrast, fludarabine suppressed ERCC1 transcription as a single agent in the majority of samples (15/20) and inhibited SJG-136-induced ERCC1 transcription when used in combination (20/20). Importantly, the ability of fludarabine to suppress ERCC1 transcription when used in combination with SJG-136 correlated with the degree of synergy observed between the two drugs (*r*^2^=0.28; *P*=0.017). This indicates that the inhibition of ERCC1 is a key mechanistic component of the synergy between the two drugs. However, samples that showed diminished fludarabine-mediated suppression of ERCC1 still retained cytotoxic synergy between the two agents pointing to additional mechanisms that contribute to their synergistic interaction. Taken together, our data demonstrate a clear relationship between SJG-136-induced DNA ICL and cytotoxicity in primary CLL cells and offer a mechanism-based rationale for the increased DNA ICL observed in samples treated with the combination of SJG-136 and fludarabine compared to SJG-136 alone. The retention of cytotoxic synergy in samples derived from patients with either *in vitro* or *in vivo* resistance to fludarabine provides a clear indication for the use of the combination of SJG-136 and fludarabine in the treatment of this group of patients.

## Figures and Tables

**Table 1:** Clinical characteristics of the CLL patients in this study

No. of patients	40
Mean age (years)	64
Sex (male/female)	25/15
Binet stage (A/B/C)	13/9/18
Previous treatment (untreated/treated)	24/16
Clinical resistance to fludarabine (resistant/sensitive)	9/16
V_H_ gene mutation (mutated/unmutated)	18/22
CD38 expression (<30%/⩾30%)	17/23
ZAP-70 expression (<20%/⩾20%)	15/25

CLL=chronic lymphocytic leukaemia.

**Table 2:** Mean drug sensitivity data for the fludarabine sensitive and fludarabine resistant subsets of CLL samples in the study

**CLL Patient samples**	**Mean fludarabine LD_50_**	**Mean SJG-136 LD_50_**	**Mean CI**
Fludarabine sensitive (*n*=20)	0.8 *μ*M±0.5 *μ*M	8.5 nM±3.0 nM	0.47±0.2
Fludarabine resistant (*n*=20)	1.7 *μ*M±0.6 *μ*M	10.2 nM±3.2 nM	0.62±0.3
*P*-value	0.004^*^	0.13	0.03^*^

CI=combination index; CLL=chronic lymphocytic leukaemia.

^*^Statistical significance at *P*<0.05 level.
